# Caracterización molecular de enterobacterias multirresistentes en dos departamentos de la selva peruana

**DOI:** 10.7705/biomedica.5720

**Published:** 2021-10-15

**Authors:** Diana León-Luna, Alexander Fajardo-Loyola, José Yareta-Yareta, Antonio Burgos-Espejo, Carlos Peralta-Siesquen, Marco Galarza-Pérez, Pool Marcos-Carbajal

**Affiliations:** 1 Facultad de Ciencias Naturales y Matemática, Universidad Nacional Federico Villarreal, Lima, Perú Universidad Nacional Federico Villarreal Universidad Nacional Federico Villarreal Lima Peru; 2 Universidad Peruana Unión, Escuela Profesional Medicina Humana, Laboratorio de Investigación en Biología Molecular, Lima, Perú Universidad Peruana Unión Universidad Peruana Unión Lima Peru; 3 Laboratorio de Referencia Nacional de Biotecnología y Biología Molecular, Instituto Nacional de Salud, Lima, Perú Instituto Nacional de Salud Lima Perú; 4 Sección de Microbiología, Hospital Regional de Pucallpa, Pucallpa, Perú Hospital Regional de Pucallpa Pucallpa Perú; 5 Laboratorio de Microbiologia, IPRESS Jorge Chávez, Madre de Dios, Perú IPRESS Jorge Chávez Madre de Dios Perú; 6 Universidad San Martín de Porres, Facultad de Medicina Humana, Centro de Investigación en Medicina Tradicional y Farmacología, Lima, Perú Universidad San Martín de Porres Lima Perú

**Keywords:** Enterobacteriaceae, farmacorresistencia microbiana, resistencia betalactámica, genes, Enterobacteriaceae, drug resistance, microbial, beta-lactam resistance, genes

## Abstract

**Introducción.:**

La aparición de enterobacterias multirresistentes y productoras de betalactamasas de espectro extendido (BLEE) en pacientes de consulta externa con infecciones urinarias, representa un problema de salud pública en Perú.

**Objetivos.:**

Caracterizar molecularmente enterobacterias multirresistentes aisladas de pacientes con diagnóstico de infección urinaria y procedentes de dos departamentos de la selva peruana.

**Materiales y métodos.:**

Se hizo un estudio descriptivo, observacional y retrospectivo de 61 aislamientos de urocultivo procedentes de la selva peruana durante 2017 y 2018. Los perfiles de resistencia se identificaron utilizando el sistema automatizado MicroScan™ y para la detección de los genes *bla*_*TEM*_ , *bla*_*CTX-M*_ , *bla*_*SHV*_ se empleó una reacción en cadena de la polimerasa (PCR) convencional.

**Resultados.:**

Las enterobacterias positivas para BLEE más frecuentes por departamento fueron *Escherichia coli* en Madre de Dios (25%, 10/40) y Ucayali (76,2%, 16/21). En ambos departamentos, el gen bla_CTX-M_ fue el más frecuente (25/61; 41), seguido por *bla*_*TEM*_ (15/61; 24,6%) y *bla*_*SHV*_ (10/61; 16,4%). En el perfil de sensibilidad antimicrobiana, se detectó 72,6% de resistencia contra ampicilina, 82,3 % contra cefalotina y 88,7% contra nitrofurantoína.

**Conclusiones.:**

El porcentaje de cepas de enterobacterias multirresistentes productoras de BLEE en ambos departamentos fue del 57,4% y el gen *bla CTX-M* fue el más frecuente.

## Abstract (en)

**Introduction.:**

The emergence of multiresistant enterobacteria producing extended- spectrum beta-lactamase (ESBL) in outpatients with urinary tract infections represents a public health problem in Perú.

**Objectives.:**

To characterize multiresistant enterobacteria isolated from patients diagnosed with urinary tract infection in two Peruvian jungle departments using molecular techniques.

**Materials and methods.:**

We conducted a descriptive, observational, and retrospective study of 61 urine culture isolates from two departments in the Peruvian jungle during 20172018. Resistance profiles were identified using the MicroScan™ automated system and a conventional polymerase chain reaction (PCR) was used for the detection of *bla*_*CTX-M*_ , *bla*_*TEM*_ and *bla*_*SHV*_ genes._._

**Results.:**

The most common positive ESBL enterobacteria for each department were *Escherichia coli* in Madre de Dios (10/40; 25%) and Ucayali (16/21; 76.2%). Gene *bla*_*CTX-M*_ was the most prevalent in both departments (25/61; 41%), followed by *bla*_*TEM*_ (15/61; 24.6%), and *bla*_*SHV*_ (10/61; 16.4%). As for the antimicrobial susceptibility profile, we detected resistance levels of 72.6% for ampicillin, 82.3% for cephalothin, and 88.7% for nitrofurantoin.

**Conclusions.:**

BLEE-producing multi-resistant enterobacteria strains in both departments were 57.4% and *bla*_*CTX-M*_ was the most common gene.

La resistencia a los antibióticos es hoy una de las mayores amenazas para la salud mundial ^1^. Las infecciones de las vías urinarias se encuentran entre las infecciones bacterianas más comunes en todo el mundo y su tratamiento constituye un reto cada vez mayor, a medida que aumentan las tasas de resistencia a los antibióticos estándar ^2^^,^^3^.

Las betalactamasas son enzimas bacterianas capaces de hidrolizar los antibióticos betalactámicos, inhibiendo su mecanismo de acción y haciéndolos inefectivos ^4^^,^^5^. Las enzimas betalactamasas de espectro extendido (BLEE) están entre las de mayor relevancia clínica e incluyen tres tipos principales: TEM, SHV y CTX-M. Hasta la fecha, se han reportado más de 400 tipos de estas enzimas y la CTX-M es la que más frecuentemente se reporta en todo el mundo ^6^.

Según los datos de la Organización Panamericana de Salud (OPS) recolectados por la Red Latinoamericana de Vigilancia de la Resistencia a los Antimicrobianos (ReLAVRA), en Latinoamérica, la resistencia a los betalactámicos ha demostrado ser más grave que en otras regiones del mundo ^7^^,^^8^^,^^9^. En Perú, por ser un país megadiverso, es necesario hacer estudios por regiones (costa, sierra y selva), porque los factores ambientales y socioeconómicos intervienen en el perfil de sensibilidad de cada agente patógeno ^10^. En la región de la selva, es necesario controlar la propagación de las bacterias multirresistentes y actualmente no se hace vigilancia epidemiológica molecular que brinde datos confiables para mitigar el problema, debido a la falta de insumos, infraestructura y recursos humanos en los laboratorios.

Los estudios se han centrado hasta ahora en la región de la costa, y es necesario hacer los análisis genotípicos de los agentes patógenos en otros establecimientos de salud de la sierra y la selva, para establecer la prevalencia y el alcance de la resistencia bacteriana, cerrar la brecha de información y proporcionar una base que oriente el tratamiento empírico ^11^.

En ese contexto, el objetivo del estudio fue caracterizar molecularmente enterobacterias multirresistentes aisladas de pacientes con diagnóstico de infección urinaria procedentes de dos departamentos de la selva peruana.

## Materiales y métodos

### Pacientes y material biológico

Se hizo un estudio descriptivo, observacional y retrospectivo en aislamientos bacterianos de muestras de orina procedentes de los hospitales públicos regionales de nivel II de Santa Rosa y Pucallpa, en los departamentos de Madre de Dios y Ucayali, respectivamente. Se analizaron 61 aislamientos de enterobacterias exclusivamente de pacientes adultos (mayores de 18 años) con diagnóstico de infección urinaria, atendidos en consulta externa durante 2017 y 2018 y procedentes de la región de selva. Se excluyeron las cepas aisladas que no fueran enterobacterias.

### Identificación bacteriana

La identificación y el perfil de sensibilidad se determinaron seleccionando las colonias en agar MacConkey mediante el uso del sistema automatizado MicroScan™ (AutoScan-4) y de paneles para bacterias Gram negativas (Dade MicroScan™), siguiendo las indicaciones del manual estandarizado ^12^. Se utilizaron 26 antimicrobianos para cada cepa y, con los valores de la concentración inhibitoria mínima (CIM), se interpretó el perfil de resistencia antibiótica según los puntos de corte recomendados por el *Clinical and Laboratory Standards Institute* 2020 ^13^. La presencia de BLEE se detectó mediante la lectura de los paneles MicroScan Type 66 (SMN:1711680) con el programa “LabPro Command Center”, los cuales constituyen un método confirmatorio de detección para las bacterias de tipo BLEE que puede personalizarse. Este método de detección utiliza la CMI de los antibióticos ceftazidima, aztreonam, cefotaxima, cefpodoxima y ceftriaxona.

Para el control de calidad de las pruebas de sensibilidad, se usaron la cepa Klebsiella pneumoniae ATCC 700603 como control positivo para la producción de BLEE y, la de *Escherichia coli* ATCC 25922, como control negativo.

### Extracción de ADN genómico

En la extracción del ADN genómico de las cepas de enterobacterias, se utilizó el método basado en columnas de silicagel del estuche innuPREP Bacteria DNA™ (Analytikjena, Alemania), con el cual fue necesario que el crecimiento bacteriano se encontrara en la fase logarítmica. El ADN aislado se almacenó a -20 °C hasta la amplificación de cada gen por PCR convencional.

### Reacción en cadena de la polimerasa

La amplificación mediante PCR se utilizó para detectar los genes BLEE (*bla*_*CTX-M*_ , *bla*_*TEM*_ , *bla*_*SHV*_ ); los iniciadores empleados fueron los descritos por Kiratisin, *et al*. ^14^, y Arce, *et al*. ^15^ (cuadro 1).

La mezcla de la reacción se ajustó a una concentración final de 25 μl según las condiciones descritas en el protocolo. La mezcla maestra fue la siguiente: 10 μl de solución tampón para PCR 10x incluido Mg (abm), 1 μl de dNTP (abm), 0,5 μl de Primer-F, 0,5 μl de Primer-R (Macrogen, South Korea), 17,3 μl de agua ultrapura (Invitrogen), 0,2 μl de Taq ADN polimerasa (abm) y 2,5 μl de ADN genómico previamente extraído de cada cepa bacteriana.

La PCR se llevó a cabo en un termociclador T100^TM^ (Thermal Cycler, BioRad, Estados Unidos) bajo las siguientes condiciones: predesnaturalización a 94 °C por 7 minutos, desnaturalización a 94 °C por 50 s, hibridación a 52-55 °C por 1 minuto, extensión a 72 °C por 60 s repetidos en 35 ciclos, y un ciclo de extensión final de 5 minutos a 72 °C y un tiempo infinito a 8 °C.

**Cuadro 1:** Iniciadores utilizados para la amplificación por reacción en cadena de la polimerasa (PCR)

Gen	Iniciadores	Secuencia	Amplicón (pba)	Referencia
blaCTX-M	CTX-M-F CTX-M-R	GAAGGTCATCAAGAAGGTGCG GCATTGCCACGCTTTTCATAG	544	13
blaSHV	SHV-F SHV-R	TGGTTATGCGTTATATTCGCC GGTTAGCGTTGCCAGTGCT	868	12
blaTEM	TEM-F TEM-R	TCCGCTCATGAGACAATAACC TTGGTCTGACAGTTACCAATGC	931	12

F: *Forward*; R: Reverse; CTX-M: cefotaximasas; TEM: temoniera; SHV: sulfhidrilo variable; pb: pares de bases; bla: genes de resistencia a betalactamasas

### Electroforesis

El producto de amplificación se analizó por electroforesis en gel de agarosa al 1,5 % (Cleaver Scientific), usando como solución tampón de carga y revelador el Safe-Green^TM^ (abm). Para verificar el tamaño del ADN amplificado, se empleó el marcador de peso molecular de 100 pb (opti DNA Marker - abm™). La corrida electroforética se llevó a 120 V por 40 minutos en el sistema de electroforesis con documentación runSTATION complete (Cleaver Scientific, United Kindom).

### Análisis estadístico

La base de datos se analizó con el programa Stata 15.0. Las medidas de frecuencia se presentan como frecuencias relativas (porcentajes). El gráfico de barras se elaboró con el programa Microsoft Excel. 2019. Por ser un estudio observacional, no requirió de variables dependientes e independientes.

### Consideraciones éticas

Los dos establecimientos de salud aprobaron el protocolo de estudio. Se garantizó la confidencialidad de los datos de los pacientes. El estudio fue evaluado y aprobado por el Comité de Ética de la Universidad Peruana Unión (N2019-CEUPeU-0001).

## Resultados

De los 61 pacientes que acudieron a consulta externa, el 65,6% (40/61) provenía del departamento de Madre de Dios (Hospital Regional de Santa Rosa) y el 34,4% (21/61) del departamento de Ucayali (Hospital Regional de Pucallpa). El 57,4% (35/61) resultó ser positivo para BLEE, y la edad media fue de 41 años. Las enterobacterias identificadas como positivas para BLEE fueron: *E. coli* con 42,6% (26/61), *K. pneumoniae* con 8,2% (5/61), *Enterobacter cloacae* con 1,6 (1/61), *Acinetobacter baumannii* con 1,6 % (1/61), *Klebsiella oxytoca* con 1,6% (1/61), y *Enterobacter aerogenes* con 1,6 % (1/61) (cuadro 2).

El gen más frecuente en los aislamientos fue el *bla*_*CTX-M*_ con 41 % (25/61) seguido por el *bla*_*TEM*_ con 24,6 % (15/61) y el *bla*_*SHV*_ con el 16,4 % (10/61). En el departamento de Madre de Dios, los genes detectados en *E. coli* fueron *bla*_*CTX-M*_ (n=6; 15 %), *bla*_*TEM*_ (n=8; 20 %) y *bla*_*SHV*_ (n=4; 10 %) y en K. pneumoniae los genes *bla*_*CTX-M*_ (n=2; 5 %), *bla*_*TEM*_ (n=2; 5 %) y *bla*_*SHV*_ (n=4, 10 %). En el departamento de Ucayali los genes detectados en *E. coli* fueron *bla*_*CTX-M*_ (n=16, 76,2 %), *bla*_*TEM*_ (n=2, 9,5 %) y *bla*_*SHV*_ (n=4, 10 %), en tanto que en K. pneumoniae no se detectó ninguno de estos (cuadro 3).

**Cuadro 2:** Frecuencia de enterobacterias BLEE y no BLEE aisladas por departamento

Enterobacterias (n=61)	Departamento de Madre de Dios (n=40)		Departamento de Ucayali (n=21)	
	BLEE + n (%)	BLEE - n (%)	BLEE + n (%)	BLEE - n (%)
*Escherichia coli*	10 (25)	20 (50)	16 (76,2)	3 (14,3)
*Klebsiella pneumoniae*	5 (12,5)	0,0	SA	SA
*Enterobacter cloacae*	1 (2,5)	3 (7,5)	SA	SA
*Acinetobacter baumannii*	1 (2,5)	0,0	SA	SA
*Klebsiella oxytoca*	SA	SA	1 (4,8)	0,0
*Enterobacter aerogenes*	SA	SA	1 (4,8)	0,0
Total	17 (42,5)	23 (57,5)	18 (85,7)	3 (14,3)

BLEE: betalactamasas de espectro extendido; SA: sin aislamiento bacteriano

**Cuadro 3:** Frecuencia de los genes de resistencia a betalactamasas detectados por enterobacteria y departamento

Enterobacterias (n=61)	Departamento de Madre de Dios (n=40)			Departamento de Ucayali (n=21)		
	bla_CTX-M_ n (%)	bla_TEM_ n (%)	bla_SHV_ n (%)	bla_CTX-M_ n (%)	bla_TEM_ n (%)	bla_SHV_ n (%)
*Escherichia coli*	6 (15)	8 (20)	4 (10)	16 (76,2)	2 (9,5	2 (9,5)
*Klebsiella pneumoniae*	2 (5)	2 (5)	4 (10)	0,0	0,0	0,0
*Enterobacter cloacae*	0,0	1 (2,5)	0,0	0,0	0,0	0,0
*Acinetobacter baumannii*	1 (2,5)	0,0	0,0	0,0	0,0	0,0
*Klebsiella oxytoca*	0,0	0,0	0,0	0,0	1 (4,8)	0,0
*Enterobacter aerogenes*	0,0	0,0	0,0	0,0	1 (4,8)	0,0
Total	9 (22,5)	11 (27,5)	8 (20)	16 (76,2)	4 (19)	2 (9,5)

Genes de resistencia a betalactamasas: *bla*_*CTX-M,*_ *bla*_*TEM*_ y *bla*_*SHV*_

**Figura 1:**
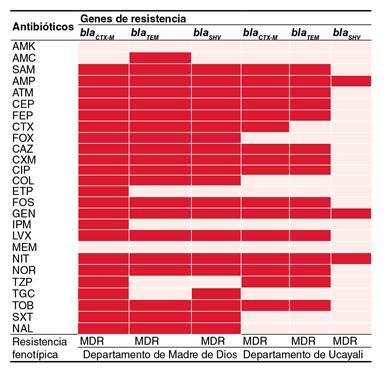
Perfil de resistencia genotípica y genes de resistencia a betalactamasas (BLEE) en enterobacterias de dos hospitales de la selva peruana

AMK: amikacina; AMC: amoxicilina con ácido clavulánico; SAM: ampicilina con sulbactam; AMP: ampicilina; ATM: aztreonam; CF: cefalotina; FEP: cefepima; CTX: cefotaxima; FOX: cefoxitina; CAZ: ceftazidima; CXM: cefuroxima; CIP: ciprofloxacina; COL: colistina; ETP: ertapenem; FOS: fosfomicina; GEN: gentamicina; IPM: imipenem; LVX: levofloxacina; MEM: meropenem; NIT: nitrofurantoína; NOR: norfloxacina; TZP: piperacilina con tazobactam; TGC: tigeciclina; TOB: tobramicina; SXT: trimetoprim sulfametoxazol; NAL: ácido nalidíxico; MDR: multirresistencia (resistente, por lo menos, a uno de dos o tres clases de antibióticos)

Para analizar la resistencia fenotípica con base en los genes *bla*_*CTX-M*_ , *bla*_*TEM*_ y *bla*_*SHV*_ , se evaluaron todos los aislamientos bacterianos positivos para BLEE y se observó, por lo menos, la expresión de uno de los genes en cada aislamiento, además, se evidenció también que otros aislamientos codificaron dos o más genes de resistencia a betalactamasas siendo estos considerados multirresistentes, como se muestra en la figura 1. En el departamento de Madre de Dios y Ucayali, se observó multirresistencia a la mayoría de antibióticos, excepto frente a amikacina (AMK) y meropenem (MEM) como es el caso de Ucayali.

**Figura 2:**
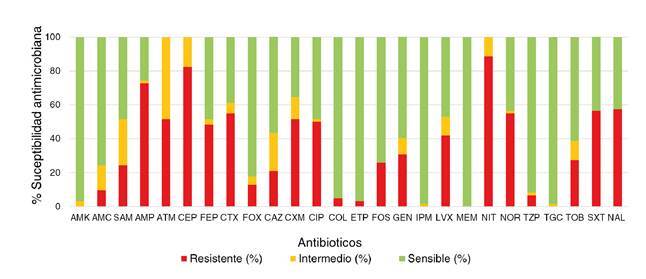
Porcentaje de sensibilidad antimicrobiana

AMK: amikacina; AMC: amoxicilina con ácido clavulánico; SAM: ampicilina con sulbactam; AMP: ampicilina; ATM: aztreonam; CF: cefalotina; FEP: cefepima; CTX: cefotaxima; FOX: cefoxitina; CAZ: ceftazidima; CXM: cefuroxima; CIP: ciprofloxacina; COL: colistina; ETP: ertapenem; FOS: fosfomicina; GEN: gentamicina; IPM: imipenem; LVX: levofloxacina; MEM: meropenem; NIT: nitrofurantoína; NOR: norfloxacina; TZP: piperacilina con tazobactam; TGC: tigeciclina; TOB: tobramicina; SXT: trimetoprim sulfametoxazol; NAL: ácido nalidíxico

Se detectaron altos porcentajes de resistencia a ampicilina (72,6%), aztreonam (51,6%), cefalotina (82,3%), cefotaxima (54,8%), cefuroxima (51,6%), ciproflaxina (50%), nitrofurantoína (88,7%), norfloxacina (54,8%) y ácido nalidíxico (57,4%). No se detectaron aislamientos resistentes a amikacina, imipenem, meropenem o tigeciclina (figura 2).

## Discusión

El propósito del estudio fue determinar la aparición de enterobacterias productoras de BLEE en los pacientes de consulta externa que acudieron a los hospitales de los departamentos de Madre de Dios y Ucayali. En el 57,4% de los aislamientos, las enterobacterias fueron productoras de BLEE, sobre todo *E. coli* y *K. pneumoniae*, lo que coincide con lo reportado por Olarte, *et al*. ^9^, y Arce, *et al*. ^15^. Morejón ^16^ señala que no solo *E. coli* y *K. pneumoniae* pueden ser productoras de BLEE, lo que concuerda con nuestro reporte, en el que se identificaron otras especies, como *E. cloacae*, *A. baumannii*, *K. oxytoca*, y *E. aerogenes*, las cuales, además, representaron el 11,4% de los aislamientos positivos para BLEE.

Según García, *et al*. ^17^, es importante resaltar la amplia distribución de los genes responsables de la resistencia a antibióticos betalactámicos en las distintas enterobacterias, así como la presencia simultánea de varios genes. En cuanto a los aislamientos, la mayoría de los estudios muestra un predominio de BLEE de tipo CTX-M en *E. coli*. En el presente estudio, el 36,1% de las cepas de *E. coli* expresó el gen bla_*CTX-M*_ y el 16,3% el bla_*TEM*_ , en tanto que el 3,3% de K. pneumoniae expresó los genes bla_*CTX-M*_ y bla_*TEM*_ ; además, se registraron los genes bla_*SHV*_ en el 9,9% de *E. coli* y el 6,6% de *K. pneumoniae*, así como la coexistencia de los tres genes: bla_*CTX-M*_ , bla_*TEM*_ y bla_*SHV*_ en el 4,9% de *E. coli*. En su estudio Galván, *et al*. ^18^, reportaron una frecuencia de 55 % del gen bla_*CTX-M*_ en y la coexistencia de bla_*CTX-M*_ y bla_TEM_ en 24% de los aislamientos de *E. coli* en orina. Por otro lado, nuestros resultados difieren de lo reportado por Castro, *et al*. ^19^ en su estudio en *E. coli* productoras de BLEE aisladas de urocultivos de pacientes de la comunidad de Chilpancingo, México, en los que registraron un 95% de bla_*TEM*_ , el más frecuente, seguido por bla_*CTX-M*_ en el 50% y bla_*SHV*_ en el 5,5%.

En cuanto a la frecuencia de los genes de resistencia a betalactamasas detectados en cada departamento, el gen *bla*_*TEM*_ representó el 20% en Madre de Dios y, el *bla*_*CTX-M*_ , el 76,2% en Ucayali. Tales registros concuerdan con lo reportado por García, *et al*. ^17^, en relación con la detección del gen bla_*CTX-M*_ en aislamientos productores de BLEE, lo cual explica suficientemente el fenotipo resistente. En el presente estudio, en el 41% de los aislamientos analizados la enzima CTX-M fue la responsable del patrón de multirresistencia, independientemente de la presencia de las enzimas de los tipos TEM y SHV, cuyos genes también fueron detectados; este porcentaje concuerda con estudios realizados en cepas de *E. coli* comunitarias a nivel mundial, con reportes de 40 a 70% de BLEE de tipo CTX-M ^20^.

Morejón ^16^ ha señalado que la cefepima puede ser efectiva frente a muchas cepas de BLEE, particularmente las SHV. Suarez, *et al*. ^21^, mencionan que la nitrofurantoína es la mejor opción en las infecciones urinarias de origen comunitario producidas por *E. coli*. En este sentido nuestro reporte difirió, ya que la prueba de sensibilidad antimicrobiana evidenció que la mayoría de las enterobacterias positivas para BLEE presentaron multirresistencia, siendo el aztreonan, la cefepima y la nitrofurantoína los antibióticos más sensibles, lo cual es una razón suficiente para abogar por el uso racional de los antimicrobianos para atenuar la velocidad del incremento o aparición de nuevas multirresistencias.

En conclusión, los resultados del estudio en los departamentos de la selva peruana señalan que la frecuencia de enterobacterias multirresistentes productoras de BLEE fue de 57,4% y que el gen bla_*CTX-M*_ fue el más frecuente. Este reporte, basado en la caracterización molecular, puede contribuir a ofrecer el tratamiento antimicrobiano correcto y, además, tiene importancia epidemiológica, ya que aporta información molecular sobre la resistencia bacteriana en la comunidad
